# Multiple synaptic connections into a single cortical pyramidal cell or interneuron in the anterior cingulate cortex of adult mice

**DOI:** 10.1186/s13041-021-00793-8

**Published:** 2021-06-03

**Authors:** Jung-Hyun Alex Lee, Zhuang Miao, Qi-Yu Chen, Xu-Hui Li, Min Zhuo

**Affiliations:** 1 Department of Physiology, Faculty of Medicine, University of Toronto, Medical Science Building, 1 King’s College Circle, Toronto, ON M5S 1A8 Canada; 2 Center for Neuron and Disease, Frontier Institute of Science and Technology, Xi’an Jiaotong University, Xi’an, 710049 Shaanxi China; 3 Institute of Brain Research, Qingdao International Academician Park, Qingdao, Shandong China

**Keywords:** Synaptic transmission, Heterogeneous excitatory input, Anterior cingulate cortex

## Abstract

The ACC is an important brain area for the processing of pain-related information. Studies of synaptic connections within the ACC provide an understanding of basic cellular and molecular mechanisms for brain functions such as pain, emotion and related cognitive functions. Previous study of ACC synaptic transmission mainly focused on presumably thalamic inputs into pyramidal cells. In the present study, we developed a new mapping technique by combining single neuron whole-cell patch-clamp recording with 64 multi-channel field potential recording (MED64) to examine the properties of excitatory inputs into a single neuron in the ACC. We found that a single patched pyramidal neuron or interneuron simultaneously received heterogeneous excitatory synaptic innervations from different subregions (ventral, dorsal, deep, and superficial layers) in the ACC. Conduction velocity is faster as stimulation distance increases in pyramidal neurons. Fast-spiking interneurons (FS-IN) show slower inactivation when compared to pyramidal neurons and regular-spiking interneurons (RS-IN) while pyramidal neurons displayed the most rapid activation. Bath application of non-competitive AMPA receptor antagonist GYKI 53655 followed by CNQX revealed that both FS-INs and RS-INs have AMPA and KA mediated components. Our studies provide a new strategy and technique for studying the network of synaptic connections.

## Introduction

It is known that cortical neurons receive multiple inputs from other cortical areas and subcortical areas [1–4]. The investigation of excitatory inputs into these cortical neurons, however, are mostly studied at a single synapse [5, 6]. Few studies have been reported for multiple inputs into a single cortical neuron at the same time. This information is critical since a single cortical neuron can receive multiple excitatory inputs at the same time in vivo. It provides the synaptic basis for convergent sensory, auditory, motor, emotional and cognitive information to the same neuron, especially for neurons in the anterior cingula cortex (ACC).

The ACC has been shown to play important roles in pain, pleasure, fear, anxiety, and other emotional and cognitive functions [7–9]. A major form of synaptic plasticity is long-term potentiation (LTP) which has been associated with the formation of a sustained state of pain via prolonged cortical excitation. This can eventually lead to a chronic pain state [10]. Studies suggest that the formation of a NMDA dependent form of LTP in response to noxious stimuli can result in the reorganization of neuronal networks, neuronal death, as well as the inability to undergo long-term depression [7, 8]. Koga et al*.* [11] was able to demonstrate that while pre-LTP is responsible for mediating the anxiety-related aspects during the induction of chronic pain, post-LTP mediates the development of chronic pain. Although our previous studies demonstrate the role of synaptic plasticity and excitatory cortical inputs in the formation of chronic pain, there is little information about the characteristic of multiple excitatory inputs into a single ACC neuron. In the present study, by combining whole-cell patch-clamp recording and 64-multi channel recording, we were able to investigate the functional connectivity between pyramidal neurons and interneurons in the ACC. We found that neurons located further away from the patched neuron in the same network have higher conduction velocities suggesting that these connections may be myelinated. Additionally, a single pyramidal neuron or interneuron can receive multiple excitatory inputs from all directions which demonstrates a high level of crosstalk between neurons located in different layers. Finally, excitatory postsynaptic currents (EPSCs) recorded in the patch-clamped pyramidal neuron or interneuron could be attenuated by the selective antagonist of AMPA/kainate (KA) receptor CNQX suggesting that the observed excitatory inputs are mainly mediated by postsynaptic AMPA/KA receptors.

## Methods

### Animals

Experiments were performed on adult male C57BL/6 mice (6–8 weeks old). Mice were purchased from Charles River Laboratories Inc. Massachusetts, United States. All mice were kept on a 12-h light/dark cycle with food and water provided ad libitum. Animal care, as well as all experiments, were conducted in accordance with the University of Toronto Animal Care Committee (UACC) guidelines for the use of experimental animals (20012315).

### Brain slice preparation

Methods used for acute brain slice preparation have been previously reported [12–14]. The mouse was deeply anesthetized with 5% isoflurane and the brain was rapidly removed and placed in oxygenated (95% O_2_ and 5% CO_2_) ice-cold artificial cerebrospinal fluid (ACSF) containing (in mM) 124 NaCl, 2.5 KCl, 2 MgSO_4_, 1 NaH_2_PO_4_, 2 CaCl_2_, 25 NaHCO_3_, and 10 d-glucose. Slices (300 μm) were cut on a Leica vibratome (VT1200S) in the high-sucrose cutting solution and immediately transferred to an ACSF-containing incubation chamber where the slices were left to recover at room temperature for 1 h. During recordings, the slices were placed in a recording chamber constantly perfused with ACSF (24–26 °C) and gassed continuously with 95% O_2_ and 5% CO_2_.

### Multi-channel probe preparation

The 64-channel multielectrode array recording system (MED64) probe (P515A, Panasonic, Japan) has an array of 64 planar microelectrodes, arranged in an 8 × 8 pattern, with an interpolar distance of 150 μm. Before use, the surface of the MED64 probe was treated with 0.1% polyethyleneimine (Sigma, St. Louis, MO; P-3143) in 25 mmol/l borate buffer (pH 8.4) overnight at room temperature according to previously reported protocols [15, 16]. The probe surface was then rinsed three times with sterile distilled water.

### Whole-cell patch-clamp/64 multi-channel recording

After 1 h for recovery in standard ACSF, slices were transferred to the recording chamber and suffused with ACSF at 28–30 °C and maintained at a 2 ml/min flow rate. The slices were positioned on the MED64 probe in such a way that the centre of the probe was closest to the central point of the ACC region.

**Fig. 1:**
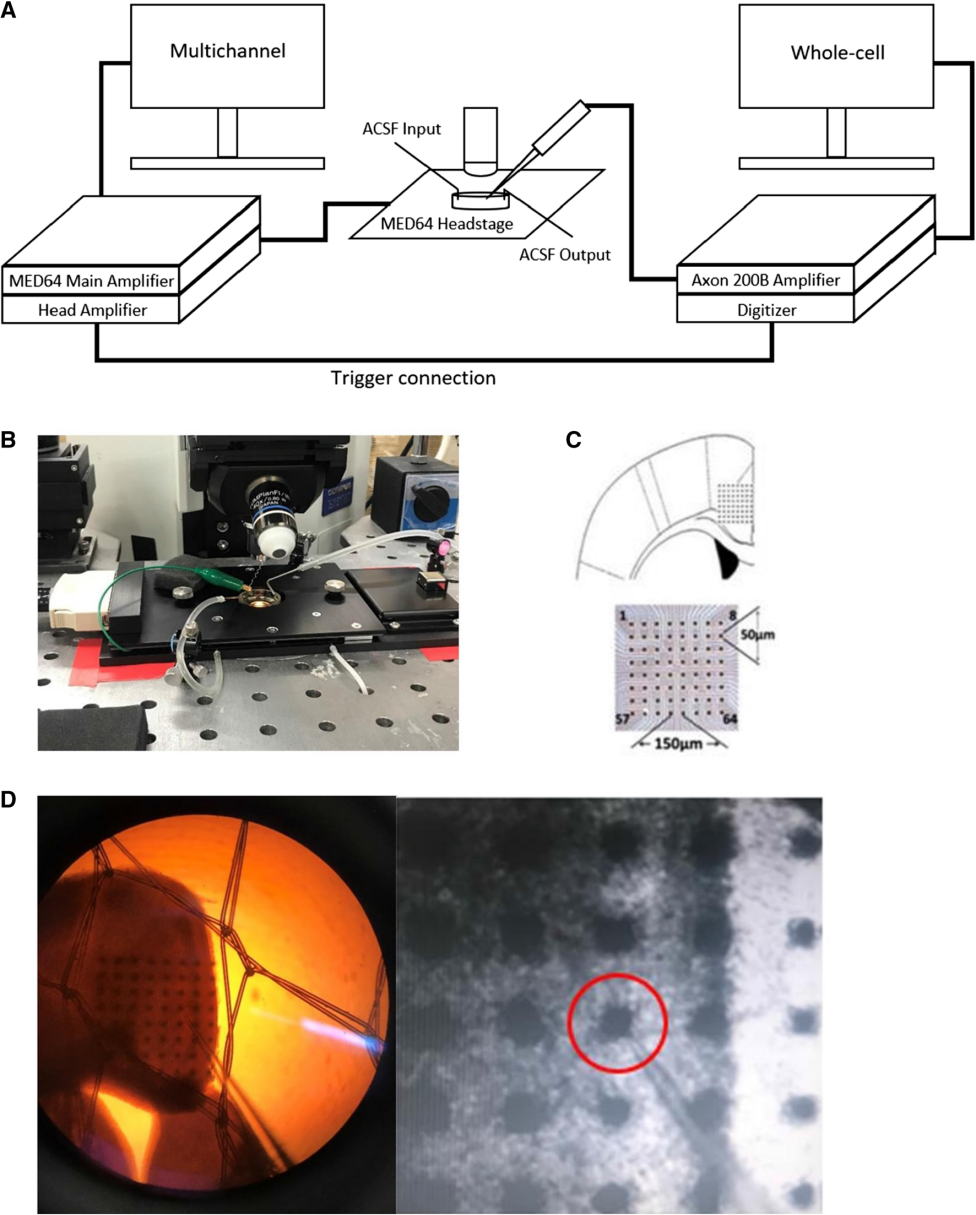
Combining whole-cell patch-clamp and MED64 multichannel systems. **A** Schematic diagram depicting how the whole-cell patch-clamp and MED64 systems are connected. A single OUT signal precedes the stimulation protocol and is sent from the MED64 head amplifier to the digitizer which triggers recording to begin in the whole cell system. **B** Photo illustrating the MED64 headstage attached onto a typical whole cell recording rig. **C** An illustration of a multichannel probe located beneath the ACC region including the dimensions. **D** A brain slice containing the ACC region is placed directly on top of the MED64 probe and a single neuron located directly above a MED64 electrode is patched (right-side image zoomed in)

Whole-cell patch-clamp recordings were performed in a recording chamber (64-channel electrode array) on the stage of a BX51WI Olympus microscope fitted with the 64-channel recording headstage. The two systems are connected by a digitizer which allows for stimulation of any of the 64 channels to trigger recording in the whole-cell patch-clamp system (Fig. 1A). Mobius software from MED64 was used in these experiments. EPSCs were mainly recorded from layer II/III neurons in the ACC using an Axopatch 200B amplifier (Axon Instruments), and the stimulations were given by a 64-channel electrode array located under the ACC region via a MED64 stimulator (Alpha Med Scientific). An initial pulse of 50.0 µA for 0.20 ms was sufficient to trigger a signal from the MED64 headstage amplifier to the axon 1322A digitizer. This was followed by a constant pulse at 0 µA for 200 ms before a secondary test pulse was used to check for a response in the patched neuron. Trace intervals were set at 30 s. The data were analyzed using Clampfit 8.0. Unless otherwise stated, the recording pipettes (3–5 MΩ) were filled with a K-gluconate internal solution (in mM); 145 K-gluconate, 5 NaCl, 1 MgCl_2_, 0.2 EGTA, 10 HEPES, 2 Mg-ATP, and 0.1 Na_3_-GTP, adjusted pH 7.3 with KOH, and osmolality of 300 mOsmol Membrane potential was held at − 70 mV to record AMPA receptor-mediated current in the presence of PTX (100 µM) and D-AP5 (50 µM). In order to record the NMDA component of the EPSC, neurons were patched using a pipette containing Cs-methanesulfonate internal solution (in mM); 145 CsMeSO_3_, 5 NaCl, 1 MgCl_2_, 0.2 EGTA, 10 HEPES, 2 Mg-ATP, 10 phosphocreatine, 0.1 Na_3_-GTP, 5 QX-314, adjusted to pH 7.2 with CsOH and osmolality of 300 mOsmol. After baseline recordings at − 70 mV, the holding potential was changed to + 40 mV in order to remove the magnesium block and record both AMPA and NMDA receptor-mediated current in the presence of PTX (100 µM) and glycine (100 nM). GYKI 53655 (50 µM), D-AP5 (50 µM), and CNQX (20 µM) were used to pharmacologically block AMPA receptors, NMDA receptors, and AMPA/kainate receptors, respectively.

### Whole-cell data analysis

Data were collected and analyzed with Clampex 8.0 and Clampfit 8.0 software (Molecular Devices). Access resistance of the patch-clamped neuron was monitored throughout the experiment, and data were discarded if the resistance changed > 15% after obtaining baseline recordings. Data representing mean values are presented with ± SEM. Statistical analysis of differences were tested by paired or un-paired two-tailed Student’s t-test, Welch t-test, and one-way ANOVA. In all cases, *p < 0.05 was considered statistically significant.

## Results

### Mapping multiple synaptic connections to a single neuron

In order to stimulate different sites in the ACC region, we replaced a typical whole-cell patch-clamp recording chamber with the MED64 probe (Fig. 1B). A brain slice was transferred into the chamber and placed so that the ACC was located above the electrodes on the MED64 probe (Fig. 1C). A single neuron located above an electrode was then located and patched (Fig. 1D). Neurons were patched typically in either layer II or III of the ACC and responded to stimulations elicited in surrounding channels up to 600 µm away. After collecting general mapping data, future neurons were only stimulated from directly neighbouring channels. In total, data from 24 neurons, 190 channels, and 23 mice were collected for whole cell experiments using MED64 probe stimulation. 16 neurons were classified as pyramidal while 8 were interneurons further separated as fast-spiking interneurons (FS INs) (n = 5) and regular-spiking interneurons (RS INs) (n = 3) based on their particular spike-patterns which have been previously reported along with their morphology [17, 18]. In order to compare observations made from single unit whole-cell recordings to evoked field excitatory postsynaptic potentials (fEPSPs) in multi-channel recordings, data from 7 neurons, 42 channels, and 7 mice were collected using a traditional 64 multi-channel setup. Based on whole-cell experiments, most of the responses from pyramidal neurons were monosynaptic, while others may be polysynaptic (n = 99 monosynaptic connections, n = 42 polysynaptic connections, total n = 141 connections). Thus, approximately 70% of the responses recorded in the patched pyramidal neuron by stimulating different channels were monosynaptic. However, stimulation of other channels using the same stimulation values and protocols led to undetectable responses which was different for each neuron. Typically, a range of 5 to 20 channels would trigger a response in the patched neuron while all other channels would result in an undetectable response.

### Excitatory responses recorded from a single pyramidal cell

**Fig. 2:**
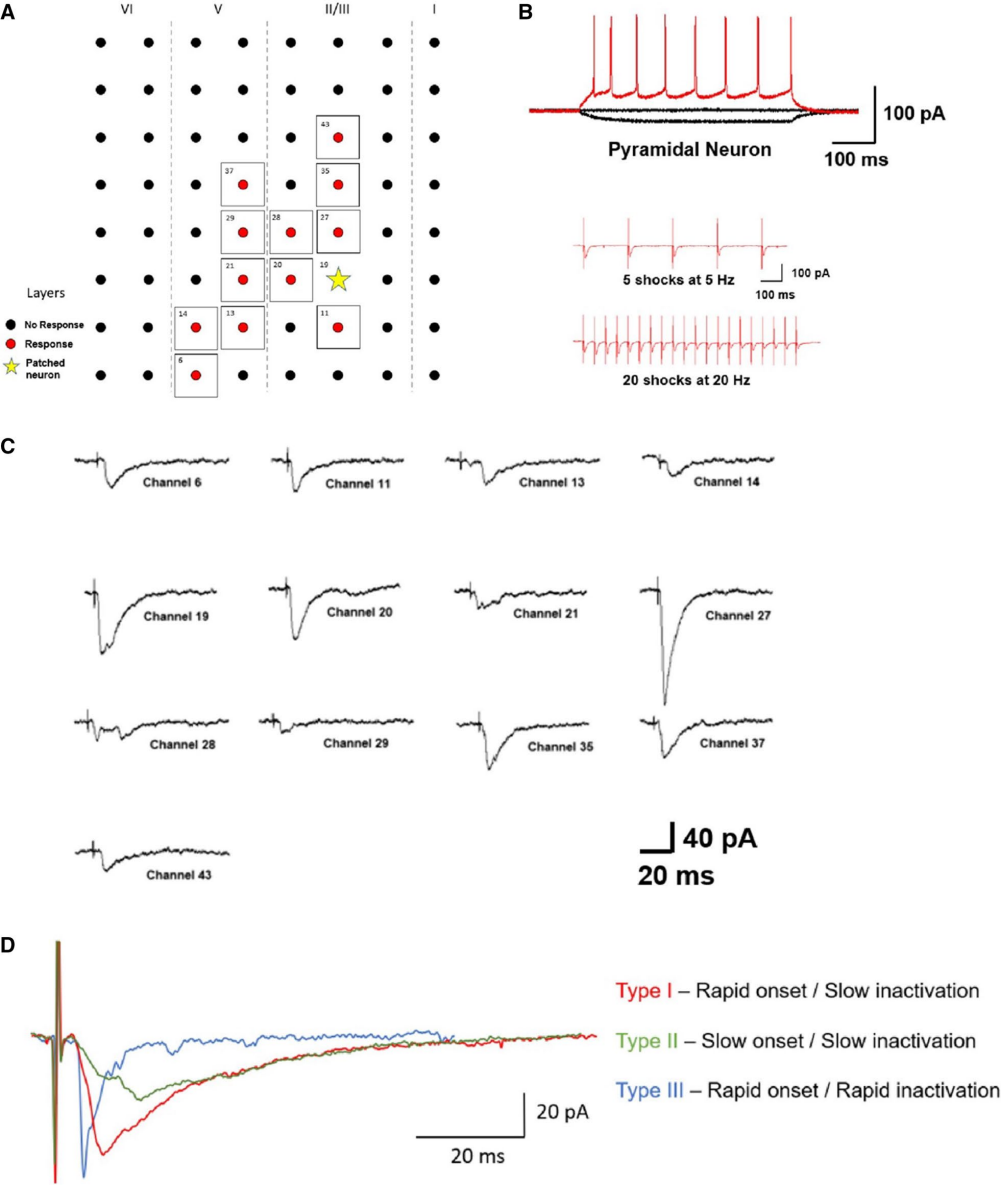
Excitatory inputs onto a single pyramidal neuron from multiple locations are not homogenous. **A** A representative mapping of a neuronal network within the ACC. The yellow star represents the patched neuron while the red dots represent channels which elicit a response in the patched neuron when stimulated. Representative traces (right) of the responses recorded in the patched neuron when stimulations were given to different channels. **B** Identification of pyramidal type by injecting step currents (− 50, 0, and 50 pA). Monosynaptic connectivity was tested using 5 shocks at 5 Hz and 20 shocks at 20 Hz. Responses triggered without failure in the presence of picrotoxin (100 µM) indicates monosynaptic connectivity. **C** Sample traces from the recorded neuron show responses with different characteristics. **D** Three representative traces are depicted to illustrate the three different types of response kinetics that were observed: Type 1—Rapid onset followed by a slow inactivation phase illustrated in red, Type II—Slow onset followed by a slow inactivation phase illustrated in green, and Type III—Rapid onset followed by rapid inactivation illustrated in blue

Pyramidal neurons located in layers II/III were primarily recorded in order to investigate neuronal connectivity in the ACC. These layers were chosen as our previous studies have characterized neurons in these areas, as well as shown them to participate in ACC related functions such as pain transmission and anxiety [19, 20]. Electrical stimulation was delivered by one of the electrodes on the 64-array located beneath the ACC. The following experiment was performed using the selective antagonist of GABA_A_ receptor picrotoxin (PTX) (100 µM). A neuron located in layers II/III were patched and neighbouring channels were stimulated in order to check which ones elicited a response in the patched neuron (Fig. 2A). The neuron was identified as a pyramidal neuron of the ACC by injecting depolarizing currents which induced repetitive action potentials with a pattern different from that of a FS- or RS-IN (Fig. 2B). In order to test whether the input responses from neighbouring channels were monosynaptic in nature, 5 shocks at 5 Hz and 20 shocks at 20 Hz were delivered. These synaptic responses followed the repetitive stimuli without failure in the presence of picrotoxin (100 µM) which suggests that these inputs are monosynaptic in nature (Fig. 2B). Different inputs from different channels led to different magnitudes of response in the patched cell as well different latencies from the time of stimulation to the time of response (Fig. 2C). In order to control the number of channels which would elicit a response across multiple slices, the smallest stimulation required to trigger a response in the patched cell was used. Recorded responses could be roughly categorized into three general types and depicted the large variation and heterogeneity between evoked EPSCs: Type I—Rapid onset/slow inactivation; Type II—Slow onset/slow inactivation; Type III—Rapid onset/rapid inactivation (Fig. 2D).

### Nerve conduction velocity in the ACC and signal distance

**Fig. 3:**
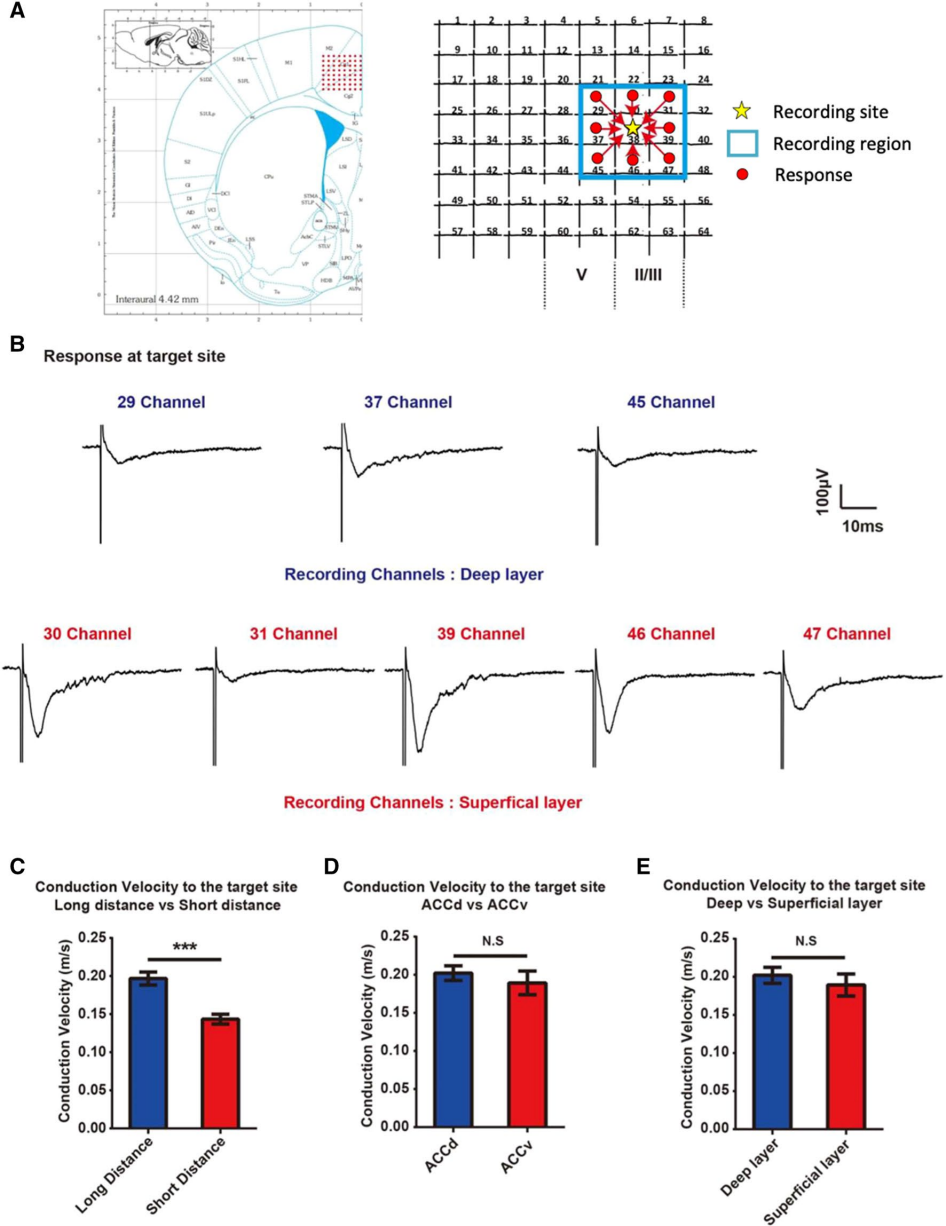
Longer-distance excitatory connections regardless of layer or region show higher conduction velocities. **A** The yellow star represents the recording site (channel 38, layer III); the red circles represent channels which elicit responses in the recording site. **B** The traces of one representative slice at target site during map recording. **C** Comparison of conduction velocity of groups with different conduction distance (the distance between stimulation site and target site). 7 mice/7 slices; Long distance group (283 µm), n = 19 channels, Short distance group, n = 23 channels. **D** Comparison of conduction velocity of groups in different regions of ACC with the same travelling distance (283 µm). Dorsal ACC (ACCd), n = 11 channels, Ventral ACC (ACCv), n = 8 channels. **E** Comparison of conduction velocity of groups in different layers with the same travelling distance (283 µm). Deep layer, n = 11 channels, Superficial layer group, n = 8 channels. ***p < 0.001, N.S. represents no significance. Error bars represent SEM

To examine the different conduction velocities of ACC fibers from the surrounding regions to the targeted neuron, we utilized the MED64 to measure the conduction latency between the stimulation site and the recording site. We then calculated the conduction velocity according to the travelling distance based on the dimensions of the MED64 probe. As shown in Fig. 3A, the arrays were placed on the ACC area based on the brain atlas as well as previous work [21, 22], and the surrounding stimulation channels (Deep layer channels: channel 29, 37 and 45; Superficial layer channels: channel 30, 31, 39, 46, 47) and the recording site (channel 38, located at superficial layer) were marked. Channel stimulation and recording were performed according to typical protocols [23, 24]. The sample traces were recorded at the target site, which show the responses induced when stimulating different ACC layers (Fig. 3B). All the analyzed channels (n = 42 channels/7 slices/7 mice) were divided into two categories based on different distances between the recording site and target site: the long-distance group (283 µm) and the short distance group (200 µm). We found that the conduction velocities in long distance group (0.20 ± 0.04 m/s, n = 19 channels) are significantly faster than those in short distance group (0.14 ± 0.03 m/s, n = 23 channels; t_40_ = 5.079, *p* < 0.001, unpaired *t* test, Fig. 3C). In other words, EPSC latency did not increase proportionately to distance from the patch-clamped neuron. However, because calculations were done using the shortest distance from stimulus electrode to patch-clamped neuron without taking into consideration actual length of connections and high variation in latency measurements, it is difficult to confidently conclude whether this is a shared trait among long distance connections. There was no significant difference in latency between long and short-distance groups (long distance group, 1.50 ± 0.08 ms; short distance group, 1.46 ± 0.07 ms; t_40_ = 0.3035, *p* = 0.7631; data not shown). The overall slow conduction velocity of both groups suggests type C unmyelinated nerve fibers, which are known to play a role in pain signaling and descending pain modulation [25–27].

### Conduction velocity between dorsal and ventral ACC

Previous studies have demonstrated that the nerve fibers in the dorsal ACC (ACCd) are more densely packed and longer than those of the ventral ACC (ACCv) [28]. To test the possibility that distinct myelination levels at specific ACC regions may influence conduction velocity, we compared the conduction velocity from both ACC dorsal and ventral sides with the same distance (283 µm). The results show that there is no significant difference of conduction speed between these two groups (ACCd group, 0.19 ± 0.02 m/s, n = 8 channels; ACCv group, 0.20 ± 0.01 m/s, n = 11 channels; t_17_ = 0.7364, *p* = 0.4716, unpaired *t* test, Fig. 3D). In addition, we also compared the conduction velocity of projections from both deep layers and superficial layers (with the same distance (283 µm) from stimulation site to response site), and the transmission speed between these two groups did not exhibit significant difference (Deep layer group, 0.20 ± 0.01 m/s, n = 11 channels; Superficial layer group, 0.19 ± 0.01 m/s, n = 8 channels; t_17_ = 0.7306, *p* = 0.4750, Fig. 3E).

### Synaptic transmission response kinetics in interneurons and pyramidal cells in the ACC

**Fig. 4:**
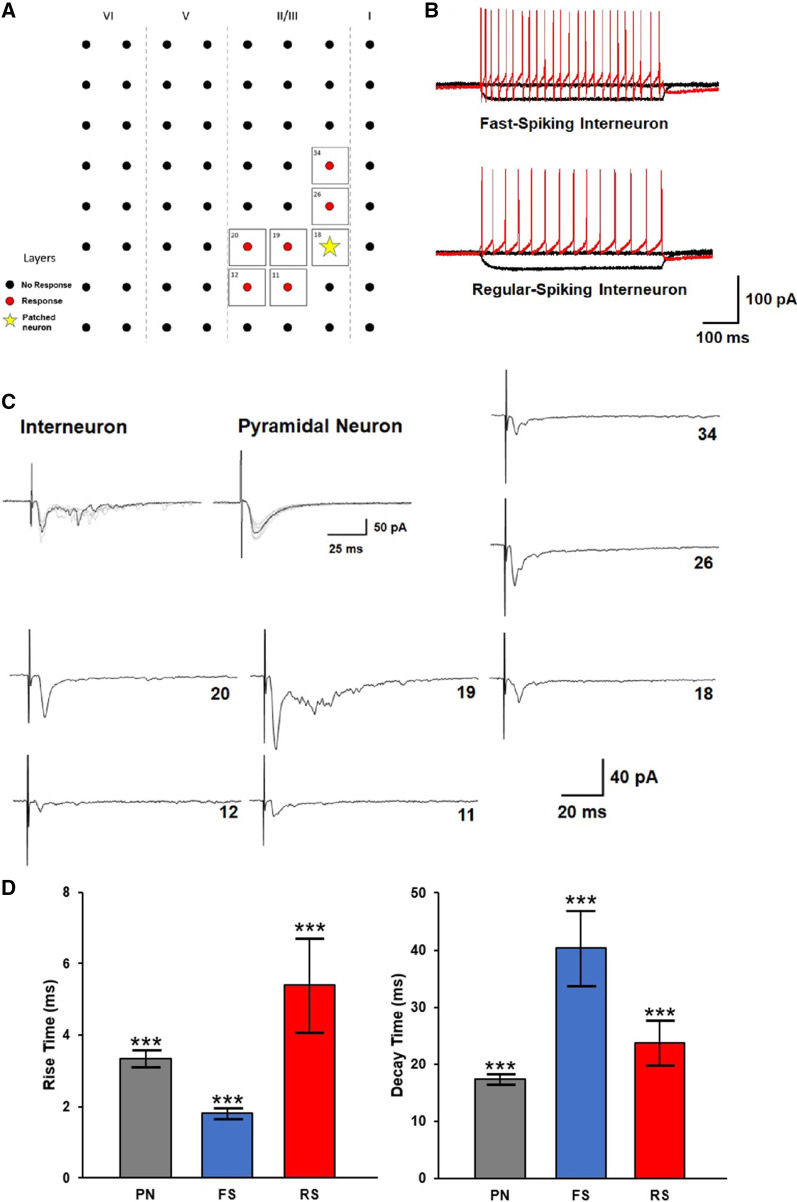
Excitatory inputs onto a single interneuron from multiple sites in the ACC. **A** Example mapping of a fast-spiking interneuron. The yellow star indicates the patched neuron and red circles denote channels which triggered a response in the patched neuron when stimulated. **B** Sample traces of action potentials recorded in fast-spiking interneurons and regular-spiking interneurons are used to determine the neuron type. **C** Two full trace samples comparing the typical response observed when an interneuron and pyramidal neuron are receiving an input. The additional traces represent the mapping data from (**A**). **D** Mean rise and decay time in different neuron types (*PN* pyramidal neuron, *FS* fast-spiking interneuron, *RS* regular-spiking interneuron) show significant difference between response kinetics for all groups (PN: n = 16 neurons/18 mice; FS: n = 5 neurons/3 mice; RS: n = 3 neurons/2 mice; ***p < 0.001 one-way ANOVA)

We recorded responses from 44 channels across eight interneurons located in layers II/III of the ACC. In terms of channel connectivity, interneurons typically respond to less channels when compared to most, but not all, pyramidal neurons. We were unable to identify any other differences in channel connectivity (Fig. 4A). Two different types of interneurons were identified based on the shape of action potentials: five fast-spiking interneurons and three regular-spiking interneurons (Fig. 4B). Similar to pyramidal neurons, multiple inputs to a single patched interneuron are not homogenous which is consistent with evidence that connections within neuronal networks are highly heterogeneous, both in structure and activity [29–31] (Fig. 4C). To see if there was a significant difference in response kinetics between neuron types, EPSCs from pyramidal neurons, fast-spiking, and regular-spiking interneurons were analyzed. Both differences in rise and decay time were found to be statistically significant between each pair across all three groups (Rise time; PN: 3.35 ± 0.24 ms; FS IN: 1.81 ± 0.16 ms; RS IN: 6.40 ± 1.32 ms, and Decay time; PN: 17.43 ± 0.93 ms; FS IN: 40.34 ± 6.60 ms; RS IN: 23.78 ± 3.90 ms, ***p < 0.001, one-way ANOVA, Fig. 4D).

**Fig. 5:**
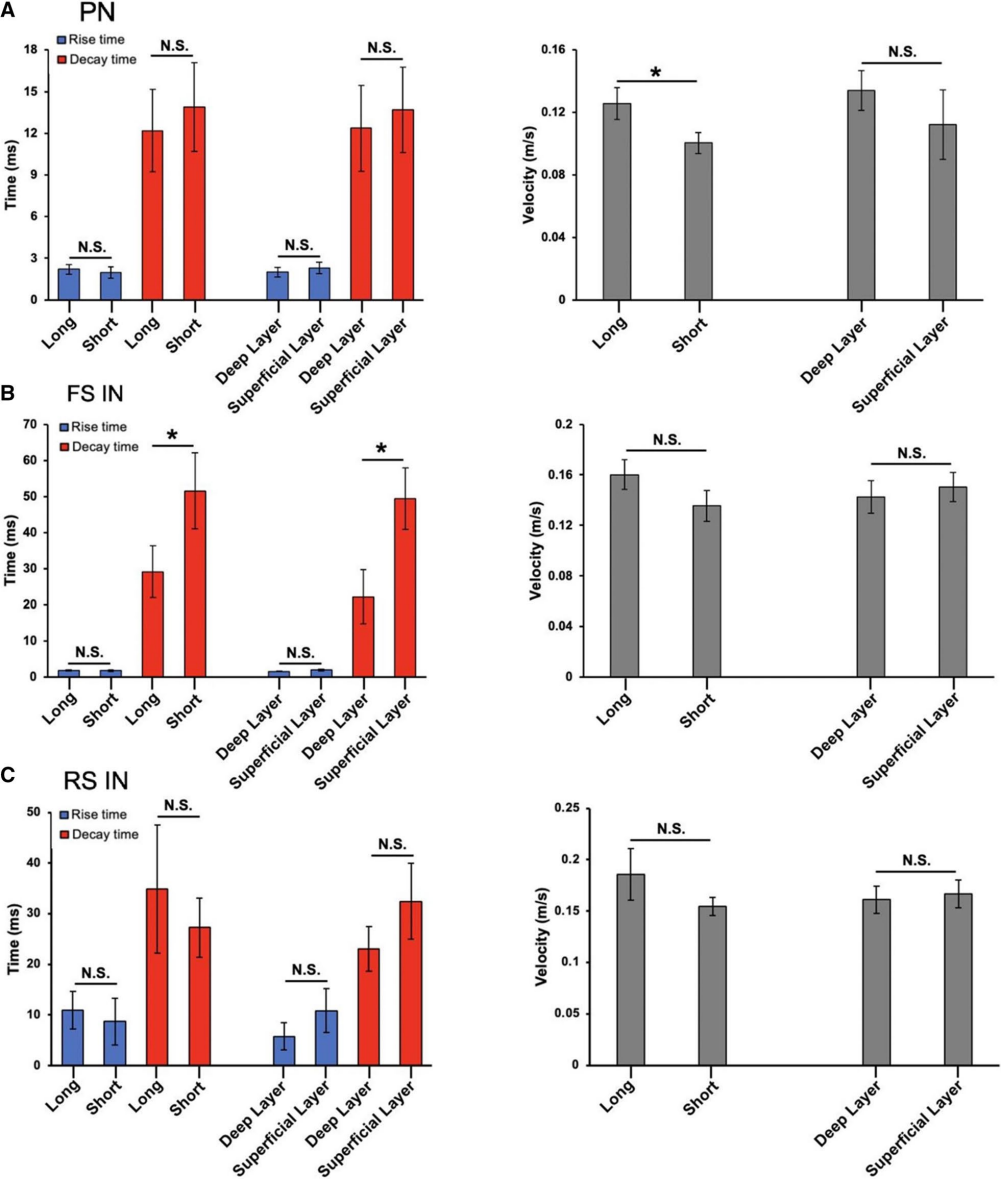
Differences in conduction velocity and response kinetics in different neuron types based on stimulation location. **A** Averaged rise and decay times based either on long distance (long) or short distance (short), and either on deep layer or superficial layer stimulation (left) and averaged conduction velocities for the same groups (right) for pyramidal neurons. There were no differences between rise and decay time when looking at long/short distance and deep/superficial layer stimulations. Conduction velocity was found to be larger in long distance stimulations in pyramidal neurons (long distance: 0.13 ± 0.007 m/s; short distance: 0.1 ± 0.01 m/s; *p < 0.05; Welch t-test). **B** Shows the same type of data was gathered for fast-spiking interneurons as (**A**) for fast-spiking interneurons. Differences in decay time between long/short distance and deep/superficial layer stimulations were found to be statistically significant (long distance: 29.1 ± 7.2 ms; short distance: 51.5 ± 10.5 ms; deep layer: 22.2 ± 7.5 ms; superficial layer: 49.4 ± 8.6; *p < 0.05; Welch t-test). Differences in rise time as well as conduction velocity between long/short distance and deep/superficial layer were found to be not significant. **C** Shows the same type of data was gathered for fast-spiking interneurons as (**A**) for regular-spiking interneurons. Differences in rise/decay times and conduction velocity when comparing long/short distance and deep/superficial layers were found to be not significant in regular-spiking interneurons. All presented data were collected under the same conditions and averaged according to their respective categories (PN: n = 45 channels/8 neurons; FS IN: n = 30 channels/4 neurons; RS IN: 18 channels/3 neurons). Error bars represent SEM

For PNs, we found that neurons located farther away from the target site on average had a higher conduction velocity than neurons closer to the target site which was found to be statistically significant [(long distance group: 0.13 ± 0.01 m/s; short distance group: 0.10 ± 0.01 m/s;* p* < 0.05, Welch* t*-test; n = 45 channels/6 mice, Fig. 5A). However, this was found to be not statistically significant when the mean conduction velocity was measured according to layer and not distance (deep layer velocity: 0.13 ± 0.01 m/s; superficial layer velocity: 0.11 ± 0.02 m/s;* p* = 0.05, Welch* t*-test, Fig. 5A)]. We also examined the rise and decay of the response observed in the patch-clamped neuron when stimulating a connected channel. Rise and decay time were calculated using Clampfit 8.0. Differences in the rise time of responses induced from deep and superficial layers were found to be statistically insignificant (deep layer group: 2.01 ms ± 0.35; superficial layer group: 2.30 ms ± 0.42, *p* = 0.3479, Welch* t*-test), as well as in the decay time (deep layer group, 12.37 ms ± 3.10, superficial layer group, 13.70 ms ± 3.07, p = 0.3819, Welch* t*-test, n = 20 channels/3 mice, Fig. 5A left). Differences in response kinetics when categorizing data by long and short distance also showed no statistical significance (long distance rise time, 2.20 ms ± 0.35, short distance rise time, 1.978 ± 0.41, *p* = 0.6851, Welch* t*-test and long-distance decay time, 12.19 ± 2.97, short-distance decay time, 13.88 ms ± 3.19, *p* = 0.7024, Welch* t*-test, Fig. 5A right). For fast-spiking interneurons, the decay time between both long/short distance as well as between deep/superficial layers was found to be statistically significant (long distance: 29.1 ± 7.2 ms; short distance: 51.5 ± 10.5 ms; deep layer: 22.2 ± 7.5 ms; superficial layer: 49.4 ± 8.6 ms; *p < 0.05; Welch* t*-test). However, there was no difference found between rise time (long distance: 1.84 ± 0.14 ms; short distance: 1.79 ± 0.28 ms; deep layer: 1.60 ± 0.15 ms; superficial layer: 1.92 ± 0.23 ms;* p* = 0.4 and 0.1 respectively, Welch* t*-test). No significant differences between conduction velocity between all groups were found (long distance: 0.16 ± 0.01 m/s; short distance: 0.14 ± 0.01 m/s; deep layer: 0.14 ± 0.01 m/s; superficial layer: 0.15 ± 0.01 m/s;* p* = 0.09 and 0.30 respectively, Welch* t*-test) (Fig. 5B). For regular-spiking interneurons, no significant differences were found in either rise/decay time and conduction velocity based on long/short distance and deep/superficial layers (long distance rise time: 10.93 ± 3.74 ms; short distance rise time: 8.73 ± 4.60 ms; long distance decay time: 34.93 ± 12.67 ms; short distance decay time: 27.23 ± 5.80 ms; deep layer rise time: 5.78 ± 2.66 ms; superficial layer rise time: 10.89 ± 4.38 ms; deep layer decay time: 23.04 ± 4.41 ms; superficial layer decay time: 32.43 ± 7.50 ms; p = 0.30, 0.30, 0.10, 0.10 respectively for each group, Welch t-test) (long distance velocity: 0.19 ± 0.02 m/s; short distance velocity: 0.15 ± 0.01 m/s; deep layer velocity: 0.16 ± 0.01 m/s; superficial layer velocity: 0.17 ± 0.01 m/s;* p* = 0.10 and 0.40 respectively, Welch* t*-test) (Fig. 5C). These data indicate that distance alone does not determine the speed of transmission to the patch-clamped neuron which is consistent with evidence that the conductance velocity of a nerve fiber is dependent on multiple factors such as the level of axon myelination, internode distance, and axon diameter [32, 33]. Comparing averaged values for rise and decay time between distance and layer indicate no difference on the population level, although individual neurons show large variation.

### Glutamate-mediated synaptic transmission

**Fig. 6:**
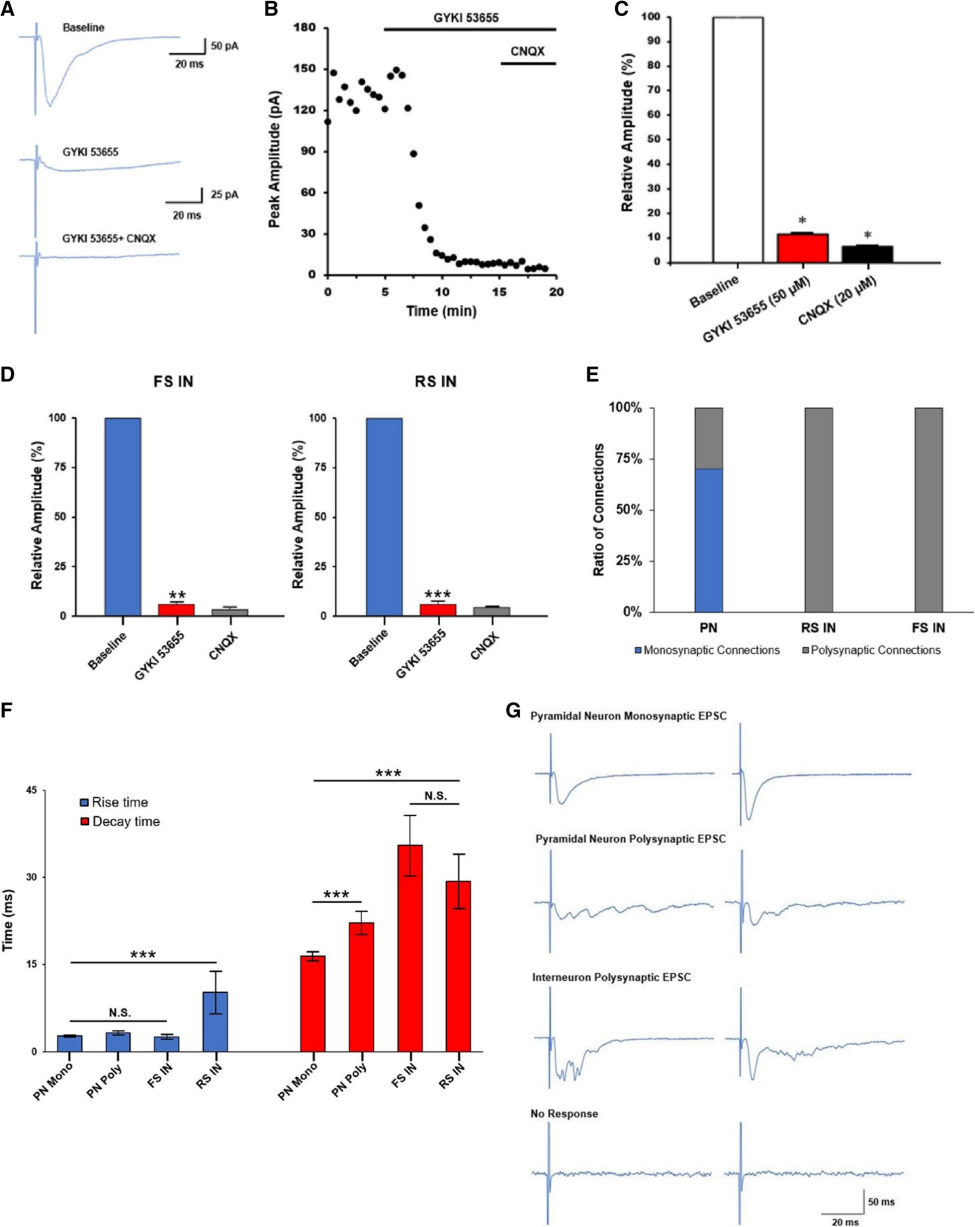
AMPA/KA receptor-mediated excitatory synaptic transmission in both pyramidal neurons and interneurons in the ACC. **A** Representative traces of a single neuron in the presence of ACSF, picrotoxin (100 µM) and AP5 (50 µM) (top). Perfusion of GYKI 53655 (50 µM) decreases EPSC amplitude and reveals KA receptor mediated EPSCs (middle). Perfusion of CNQX (20 µM) blocked all residual currents (bottom). **B** Plotting the time course of GYKI 53655 and CNQX perfusion and its effect on EPSCs recorded in the patched neuron. **C** Statistical results comparing the percentage of EPSCs during baseline, GYKI, and GYKI + CNQX perfusion (41 channels, n = 6 neurons/3 mice). Differences in percent amplitude were found to be significant *p < 0.05, SEM is represented by error bars. **D** Pharmacological isolation of AMPA and KA receptors on interneurons using GYKI 53655 (50 µM) and CNQX (20 µM) on both FS-INs and RS-INs shown as a percent of the baseline amplitude. In FS-INs, GYKI 53655 resulted in a 94.3 ± 1.4% decrease from 105.5 ± 17.3 pA to 6.1 ± 0.6 pA (**p < 0.01). In RS-INs, GYKI 53655 resulted in a 94.2 ± 1.6% decrease from 60.3 ± 10.6 pA to 3.5 ± 0.4 pA (***p < 0.001). **E** A bar graph showing the proportion of mono to polysynaptic connections in pyramidal neurons, FS, and RS interneurons (70.2% monosynaptic; n = 16, 5, and 3 neurons respectively, 24 mice total). **F** A bar graph re-examining response kinetics of neurons based on neuron type and not distance shows that rise time is significantly larger only in regulars-spiking interneurons (Rise time; RS IN: 10.2 ± 3.6 ms; PN Mono: 2.7 ± 0.1 ms; PN Poly: 3.3 ± 0.3 ms; FS IN: 2.6 ± 0.4 ms; ***p < 0.001; one-way ANOVA). The shortest average decay time was observed in monosynaptic pyramidal neurons followed by polysynaptic pyramidal neurons (***p < 0.001; Welch t-test). Both fast-spiking and regular-spiking interneurons had larger decay times than that of pyramidal neurons (Decay time; PN Mono: 16.4 ± 0.7 ms; PN Poly: 22.2 ± 1.9 ms; FS IN: 35.5 ± 5.2; RS IN: 29.3 ± 4.7; ***p < 0.001; one-way ANOVA). **G** Sample traces depicting mono and polysynaptic responses in pyramidal neurons, polysynaptic responses in interneurons, and typical undetectable responses

We investigated which receptors may be involved in the transmission of synaptic responses within these neuronal networks. Baseline recordings in PNs were performed in the presence of picrotoxin (100 µM) and D-AP5 (50 µM) (Fig. 6A top). This was followed by GYKI 53655 (50 µM) perfusion resulting in a decrease in recorded EPSC amplitude. Because GYKI is an AMPA receptor antagonist, residual EPSCs recorded were considered to be mediated by KA receptors [34] (Fig. 6A middle). Next, perfusion of CNQX (20 µM), a competitive antagonist of both AMPA and KA receptors, was able to block residual currents in all channels (Fig. 6A bottom). A single channel was chosen from three mapping recordings to observe the effect of both GYKI 53655 and CNQX over a time course. The average EPSC amplitude of AMPA/KA EPSCs from these recordings were: 133.3 ± 12.3 pA; KA EPSCs: 8.7 ± 0.5 pA, n = 3 neurons/3 mice (Fig. 6B). Relative to the baseline EPSCs consisting of both AMPA/KA mediated EPSCs, KA EPSCs was 11.5 ± 2.9% which was blocked by CNQX (total averaged AMPA/KA EPSCs: 75.2 ± 16.3 pA; KA EPSCs: 7.6 ± 2.0 pA, n = 41 channels, 6 neurons/3 mice) (Fig. 6C). The same procedure was also used to measure baseline response followed by drug perfusion in FS INs and RS INs. In FS-INs, GYKI 53655 resulted in a 94.3 ± 1.4% decrease from 105.5 ± 17.3 pA to 6.1 ± 0.6 pA (**p < 0.01). In RS-INs, GYKI 53655 resulted in a 94.2 ± 1.6% decrease from 60.3 ± 10.6 pA to 3.5 ± 0.4 pA (***p < 0.001) (Fig. 6D). The data shows that the degree of EPSC peak amplitude decrease after application of GYKI 53655 is not the same across all channels suggesting variability of receptor composition located on different synapses.

**Fig. 7:**
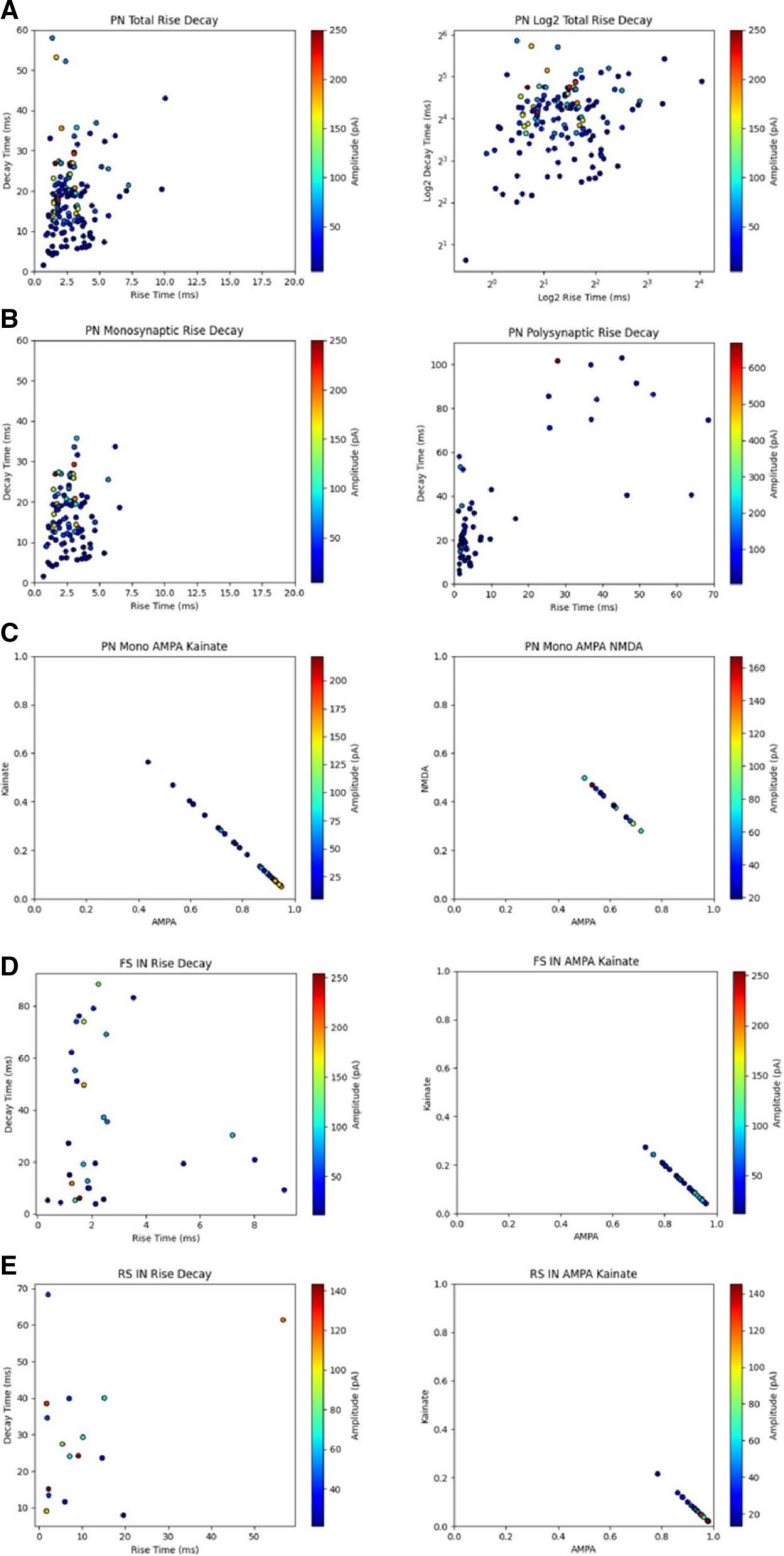
Distribution of kinetics and receptor ratios in pyramidal neurons and interneurons in the ACC. **A** A 3-variable distribution plot illustrating the spread of rise and decay time kinetics with peak amplitude in all pyramidal cells (left) and the same plot over a log2 scale for better visualization (n = 24 neurons, 23 mice, 190 channels). **B** A rise and decay time plot for only monosynaptic responses (left; n = 16 neurons) and polysynaptic responses (right; n = 8 neurons). **C** A plot showing the ratio of AMPA to KA receptors which contribute to monosynaptic responses (left; n = 8 neurons, 6 mice, 110 channels) and the same plot showing the AMPA to NMDA receptor ratio (right; n = 5 neurons, 3 mice, 28 channels). **D** A 3-variable distribution plot illustrating the spread of rise and decay time kinetics with peak amplitude (left) and a 3-variable distribution plot illustrating the ratio of AMPA to KA receptors with peak amplitude (right) for FS-INs (n = 42 channels, 3 mice). **E** Illustrates the same type of data was gathered for fast-spiking interneurons as (**C**) with RS-INs (n = 20 channels, 2 mice). Both FS INs and RS INs have statistically higher decay times than pyramidal neurons and while FS INs show the highest decay time, it is statistically insignificant relative to RS INs (PN Mono: 16.4 ± 0.7 ms; PN Poly: 22.2 ± 1.9 ms; FS IN: 35.5 ± 5.2 ms; RS IN: 29.6 ± 4.7 ms; **p < 0.01; one-way ANOVA; error bars represent SEM)

In order to better visualize the spread of rise and decay time, data were split between monosynaptic and polysynaptic connections and reanalysed (Fig. 6E–G) before the recorded values for individual neurons were mapped on a distribution plot, color-graded to show peak EPSC amplitude. The values for rise time ranged from approximately 0.7 to 10 ms and 1.5 to 58 ms for decay time, representing a large variation in response kinetics observed from the patch-clamped neuron. Rise time values below approximately 3.2 ms and decay time values after 10 ms were associated with higher peak EPSC amplitudes (Fig. 7A). To see if there were any differences in the spread of data when separating monosynaptic and polysynaptic responses, we replotted the data and found that monosynaptic responses have rise and decay time values within a much narrower range compared to that of polysynaptic responses which are spread over a larger area (Fig. 7B). In a subset of experiments, baseline EPSCs were recorded in the presence of PTX and D-AP5, an NMDA receptor antagonist, followed by bath application of GYKI 53655, a non-competitive AMPA receptor antagonist, which attenuated most of the recorded responses. Remaining current was completely blocked by CNQX, suggesting that the receptors responsible for the remaining current were KA receptors excluding GluK3 subunits, which is also blocked by GYKI 53655. This allowed us to plot the ratio between AMPA and KA receptor-mediated responses showing that AMPA receptor-mediated responses composed majority of the response in most of the neurons and that a higher AMPA receptor component was associated with higher peak EPSC amplitude (Fig. 7C left). In a similar experiment, we recorded baseline EPSCs in the presence of PTX and glycine, a co-agonist required for NMDA receptor activation, followed by a change in membrane holding potential to record combined AMPA and NMDA receptor-mediated responses. Application of D-AP5 was able to isolate the AMPA receptor component allowing us to plot the ratio between AMPA and NMDA receptor-mediated responses indicating an average 3:2 ratio (Fig. 7C right). The same approach was used for both FS INs and RS INs which showed that the AMPA receptor-mediated component was much higher than the KA receptor component in the same group as well as what was observed in PNs (Fig. 7D, E). These data suggest possible differences in expression of receptor type and number between neurons types as well as across synapses of the same neuron. Differences in measured ratios of different receptor types observed in the same patch-clamped neuron is likely explained by the high level of molecular diversity of glutamatergic and GABAergic synapses located throughout the brain [35, 36].

## Discussion

In the present study, we report that a single pyramidal or interneuron can receive multiple, heterogenous inputs resulting in different postsynaptic responses. Analysis of recorded EPSCs show differences in rise and decay time, as well as peak amplitude. Pharmacological isolation of AMPA, KA, and NMDA EPSC components show varying ratios in the same neuron which is likely a result of different synaptic activation based on stimulation site. Further analysis of the latency from stimulation artifact to EPSC and stimulation electrode distance allowed us to approximate conduction velocity values which resembled slow, unmyelinated type C fibers. To our knowledge, this is the first report of mapping multiple inputs onto a single pyramidal neuron or interneuron in the ACC using in vitro whole-cell patch-clamp recording integrated with 64-channel stimulation. The key feature of this technique is the ability to record different responses in a single neuron using stimulation regions at fixed distances. This method can be used to visualize the connectivity of a patch-clamped neuron which can provide more information regarding synaptic transmission characteristics in target brain regions. Furthermore, this method will help to study synaptic plasticity on the level of neuron populations in future studies.

Although common models of the neuron tend to simplify and group multiple dendritic inputs into a single input signal, it is well known that neurons can receive diversified patterns of inputs [37]. Both structural and statistical modelling data provide evidence for the presence and role of multisynaptic connections, some of which may be highly redundant. This neural redundancy has been hypothesized to be a mechanism for neural stability and recovery from damage which highlights the importance of multisynaptic connections [38]. Neurons contain thousands of synapses across multiple dendritic branches and integrate multiple, presumably heterogeneous signals, into a single recordable EPSC at the level of the cell body before diversification of signal output at numerous axon terminals. Varying ratios and density of glutamatergic receptors at different synapses also help explain the range of AMPA and NMDA components [39]. Furthermore, probabilistic models of neuronal connectivity show that neurons are able to detect and recognize different input patterns which serve as a robust mechanism underlying sequential memory formation [40, 41]. Although we cannot isolate individual input signals using this set-up, direct stimulation of different channels on the 64-channel electrode array result in EPSCs with varying kinetics in the same postsynaptic neuron. However, a dual patch-clamp method has been used to study the various excitatory or inhibitory synaptic inputs into either pyramidal or interneurons in the ACC, as well as to report reciprocal connections between neuron types which suggest highly complex neuronal microcircuitry [42]. In physiological conditions, it is likely that a postsynaptic neuron can receive a mixture of excitatory and inhibitory inputs during typical activity which would indicate the importance of excitation-inhibition balance in synaptic transmission. Thus, we conclude that recorded EPSCs in the postsynaptic neuron are a result of distinct patterns of synaptic input combinations. Functionally, the different possible patterns of dendritic activation serve as a likely mechanism for the encoding of learning and memory. Because the ACC has been previously reported of being involved in pain transmission and processing, as well as having reciprocal projections to the amygdala and prefrontal cortex, this mechanism may also help explain the formation of chronic pain and its negative effects on daily life.

There are three general classifications of nerve fibers both in central and periphery neurons which are associated with different types of sensory receptors or muscle fibers. Type A and B fibers are myelinated and transmit signals rapidly. In contrast, type C fibers are unmyelinated, have reportedly much slower conduction velocities, and have been identified in playing a role in the transmission of pain-related information [43, 44]. There have been very few studies regarding the conductance velocities of nerve fibers in the central nervous system (CNS) as most of the information we know about conductance velocities come from the peripheral nervous system (PNS) [45, 46]. However, a previous study focusing on thalamocortical projections utilizing whole-cell patch-clamp techniques reported certain conduction velocity values similar to what we observed in the ACC [47]. Our data shows that the conduction velocity of the activated afferent fibers fall under the range of unmyelinated type C fibers; although we cannot rule out the possibility that higher values of conduction velocities may be observed under in vivo conditions as it rules out the possibility of deterioration of nerve fibers. Other variations in synaptic transmission may be the result of highly diverse cell type expression and density in different cortical layers. For example, neocortex layer I is relatively low in overall cell density when compared to other layers but contains a large number of GABAergic neurons involved in the inhibitory modulation of local cortical networks [48]. Therefore, the stimulation of different channels in different layers likely triggers the activity of neuron populations with varying cell type compositions which would impact synaptic transmission and the postsynaptic response.

Due to their slower conduction rates, type C fibers are believed to be responsible for the slow and long-lasting spread of pain. Previous studies indicate that the activation of C fibers in the dorsal horn of the spine leads to temporal summation of secondary pain (TSSP) which can activate brain regions such as the ACC and thalamus [49]. It is possible that such TSPP may also take place in the brain regions that related to pain perception. Future studies are clearly needed to investigate molecular and synaptic mechanism of such effect. Considering stimulation or inhibition of the ACC neurons have also been shown to have descending modulatory effects on the degree of potentiation of spinal excitatory synaptic transmission [50], it is likely that injury related cortical excitation may be encoded in the ACC via distinct activation patterns of multiple synaptic inputs leading to persistent chronic pain state in response to C-fiber activation.

## Data Availability

Please contact author for data requests.
